# The Escape of Cancer from T Cell-Mediated Immune Surveillance: HLA Class I Loss and Tumor Tissue Architecture

**DOI:** 10.3390/vaccines5010007

**Published:** 2017-02-27

**Authors:** Federico Garrido, Francisco Perea, Mónica Bernal, Abel Sánchez-Palencia, Natalia Aptsiauri, Francisco Ruiz-Cabello

**Affiliations:** 1 Servicio de Analisis Clinicos e Inmunologia, UGC Laboratorio Clinico, Hospital Universitario Virgen de las Nieves, Granada 18014, Spain; franjapega@correo.ugr.es (F.P.); monicabernals@hotmail.com (M.B.); fruizc@ugr.es (F.R.-C.); 2 Instituto de Investigacion Biosanitaria ibs.Granda, Granada 18014, Spain; naptsiauri@ugr.es; 3 Departamento de Bioquimica, Biologia Molecular e Inmunologia III, Facultad de Medicina, Universidad de Granada, Granada 18071, Spain; 4 Unidad de Cirugía Torácica, Hospital Universitario Virgen de las Nieves, Granada 18014, Spain; abelsapara@hotmail.com

**Keywords:** HLA class I loss, tumor infiltrating lymphocytes (TILs), tumor immune escape

## Abstract

Tumor immune escape is associated with the loss of tumor HLA class I (HLA-I) expression commonly found in malignant cells. Accumulating evidence suggests that the efficacy of immunotherapy depends on the expression levels of HLA class I molecules on tumors cells. It also depends on the molecular mechanism underlying the loss of HLA expression, which could be reversible/“soft” or irreversible/“hard” due to genetic alterations in HLA, β2-microglobulin or IFN genes. Immune selection of HLA-I negative tumor cells harboring structural/irreversible alterations has been demonstrated after immunotherapy in cancer patients and in experimental cancer models. Here, we summarize recent findings indicating that tumor HLA-I loss also correlates with a reduced intra-tumor T cell infiltration and with a specific reorganization of tumor tissue. T cell immune selection of HLA-I negative tumors results in a clear separation between the stroma and the tumor parenchyma with leucocytes, macrophages and other mononuclear cells restrained outside the tumor mass. Better understanding of the structural and functional changes taking place in the tumor microenvironment may help to overcome cancer immune escape and improve the efficacy of different immunotherapeutic strategies. We also underline the urgent need for designing strategies to enhance tumor HLA class I expression that could improve tumor rejection by cytotoxic T-lymphocytes (CTL).

## 1. Introduction

There is a general consensus among immunologists that tumor cells can be recognized and destroyed by CD8+ T lymphocytes in vitro and in vivo. Data obtained from different groups support this concept [1,2,3]. Tumor antigens are presented to CTLs as small peptides via Major Histocompatibility Complex class I molecules (MHC-I, or HLA-I in humans) [4]. It is likely that T cell-mediated immune surveillance is, in fact, destroying nascent tumors during our life time without any noticeable signs of the rejection process. Nevertheless, tumors grow and metastasize without any visible immune control. Different tumor-immune escape mechanisms have been described that could explain the paradox of the absence of a CTL-mediated tumor rejection and the existence of tumor peptides recognized by CD8+ T cells [5].

HLA-I loss or downregulation is one of the tumor escape mechanisms widely used by cancer cells to evade T cell-mediated immune destruction [6,7,8,9]. Our laboratory has been analyzing in detail different HLA-I-altered phenotypes in human tumors [10] and we have proposed that the molecular mechanism responsible for the HLA-I loss predetermines the possibility to recover normal HLA-I expression and the success of T cell-mediated immunotherapy [11,12]. When the underlying molecular mechanism is reversible, any immunotherapy may activate the release of TH1 type cytokines in the tumor microenvironment, leading to upregulation of HLA-I expression and facilitating tumor rejection. In contrast, if the molecular mechanism is irreversible/“hard”, caused by mutations or Loss of Heterozygosity (LOH) in chromosomes 6 or 15, tumor cells are likely to remain HLA-I negative and subsequently escape immune rejection. This review summarizes these findings, focusing on the newly described association between tumor “rejection” or “escape” phenotypes and corresponding changes observed in tumor tissue architecture.

## 2. Tumor Immune Escape and HLA Class I Loss

Evidence obtained from different laboratories, including ours, indicate that HLA-I loss is a frequent finding in human primary tumors and in metastases [13,14]. Due to the complexity of the HLA genetic system and the difficulties in measuring the HLA-I expression in human tissue, the available information on tumor HLA-I expression does not always reflect the real pattern and the character of HLA-I alterations [15]. Additional detailed analysis is required to obtain a more accurate picture of HLA-I defects and the underlying molecular mechanisms in a given type of cancer. It is important to keep in mind that initially most tumors are HLA-I positive and the specific T cell-mediated immune reaction should be able to destroy these aberrant cells [13]. This process, called “T-cell immune selection”, generates tumors with a different ratio of HLA-I positive and -negative cancer cells depending on the strength of the immune response [16]. In 1996, our laboratory organized an “HLA and Cancer” component of the International HLA workshop [9]. One of the major tasks of the component was to establish criteria to classify HLA expression in tumor tissues as HLA-I positive (more that 75% cells stained), heterogeneous (between 25% and 75% of cells stained), and HLA-I negative (less than 25% HLA-I positive tumor cells). This tumor classification is in concordance with a progressive immune selection of HLA-I negative tumor variants with successive formation of tumor lesions mainly composed of HLA-I negative cancer cells [17]. In this context, published observations that HLA-I expression in pancreatic and lung cancer inversely correlates with the degree of tumor infiltration with lymphocytes, suggests an active T cell-mediated immune selection in human tumors [18,19].

## 3. T Cell Immune Selection of MHC Class I Negative Tumor Variants

As indicated earlier, T cell-mediated immune response is an important mechanism of the destruction of HLA-I positive tumor cells [20]. At the same time, this response is also the mechanism of selection of HLA-I negative tumor variants. There is accumulating evidence that T cell immune selection certainly plays an important role in the outgrowth of the MHC/HLA-I negative tumor variants [16,17,21]. We have previously reported that a mouse fibrosarcoma tumor clone (which is MHC-I negative in baseline conditions, but inducible by IFN-gamma), produces “MHC-I positive” spontaneous lung metastasis in immunodeficient nude/nude mice lacking T-cell, while in syngeneic immunocompetent mice it generates “MHC-I negative” metastases [22]. In other mouse models, it has been demonstrated that positive tumor MHC-I expression correlates with tumor immunogenicity and serves as a key predictive marker of the response to immunotherapy [23].

## 4. Immunotherapy Selects Tumor Cells with Irreversible/“Hard” HLA-I Lesions

**Figure 1:**
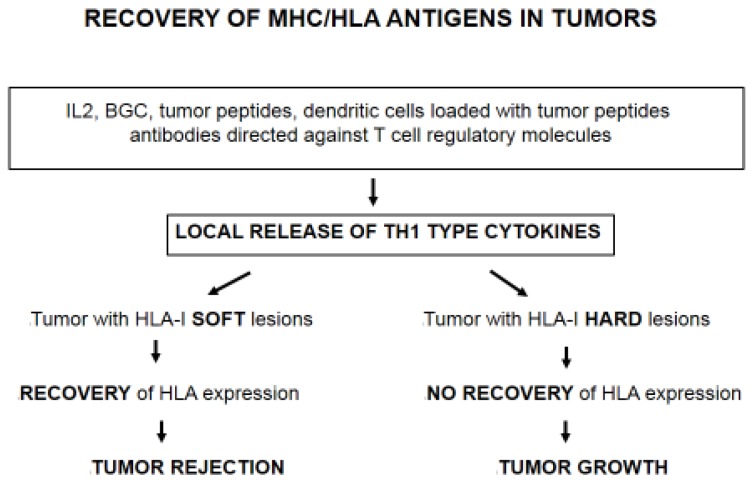
Recovery of major histocompatibility complex (MHC)/HLA-I antigens in tumors. Different treatments, including IL-2, Bacillus Calmette–Guérin (BCG), or tumor peptides, can boost anti-tumor T cell-mediated responses. Currently, monoclonal antibodies against “immune checkpoint” molecules are also being actively used in the clinic. All these therapies can modify the tumor microenvironment and induce the release of TH1 type cytokines. HLA-I deficient tumors can upregulate HLA-I expression depending on the nature of the underlying molecular alteration. If the defect is reversible/“soft”, tumor or metastatic lesion can be rejected after cytokine-mediated recovery of the antigen presentation capacity. If the alteration is irreversible/“hard”, tumor or metastasis will remain HLA-I negative and, most likely, will escape T cell responses and continue to grow.

The analysis of different subcutaneous metastases obtained from a melanoma patient with mixed response to immunotherapy showed that regression or progression of metastatic lesions is associated with the presence of HLA-I molecules on the tumor cell surface [24,25,26]. Melanoma lesions with high HLA-I expression level were heavily infiltrated by CD4+ and CD8 positive T cells and were rejected. In contrast, metastatic lesions with low HLA-I expression were resistant to immunotherapy and progressed. These findings strongly suggest that immunotherapy induces tumor HLA-I upregulation and rejection of cancer cells with “reversible/soft lesions” [12] (Figure 1). Similar results were obtained in spontaneous metastases induced in a mouse fibrosarcoma [22]. In this context, it has been recently reported that the recurrent lesions in four melanoma metastatic patients treated with monoclonal antibodies directed against “immune checkpoint” molecules harbor mutations in β2-microglobulin and IFN genes, confirming the idea that different types of immunotherapy can activate T cell-mediated immune response and select tumor cells with irreversible/“hard” lesions [27] (Figure 1). IFNs and other TH1 type cytokines undoubtedly play a major role in the upregulation of tumor MHC-I expression and in the activation of antigen presentation, ultimately leading to tumor rejection [28,29].

## 5. Changes in Stromal/Tumor Tissue Organization and HLA Class I Loss

We have recently obtained evidence indicating that tumor tissue architecture changes dramatically with HLA-I loss during cancer progression [13,18]. In lung cancer HLA-I expression strongly correlates with lymphocyte/macrophage infiltration pattern and tumor tissue organization reflecting how an immune-privileged compartment with ineffective anti-tumor immunity is generated during cancer development. These changes also affect the pattern of leucocyte infiltration and its localization within or outside the tumor area. We have classified these phases or types of tumor tissue organization as follows: permissive Phase I with HLA-I positive/heterogeneous expression pattern and tumor infiltrating lymphocytes (TILs); and encapsulated/non-permissive Phase II with HLA-I negative tumor cells and peritumoral localization of leucocytes and other immune cells [18].

### 5.1. A Permissive Tumor Tissue Structure Observed in HLA-I Positive Tumors (Phase I)

**Figure 2:**
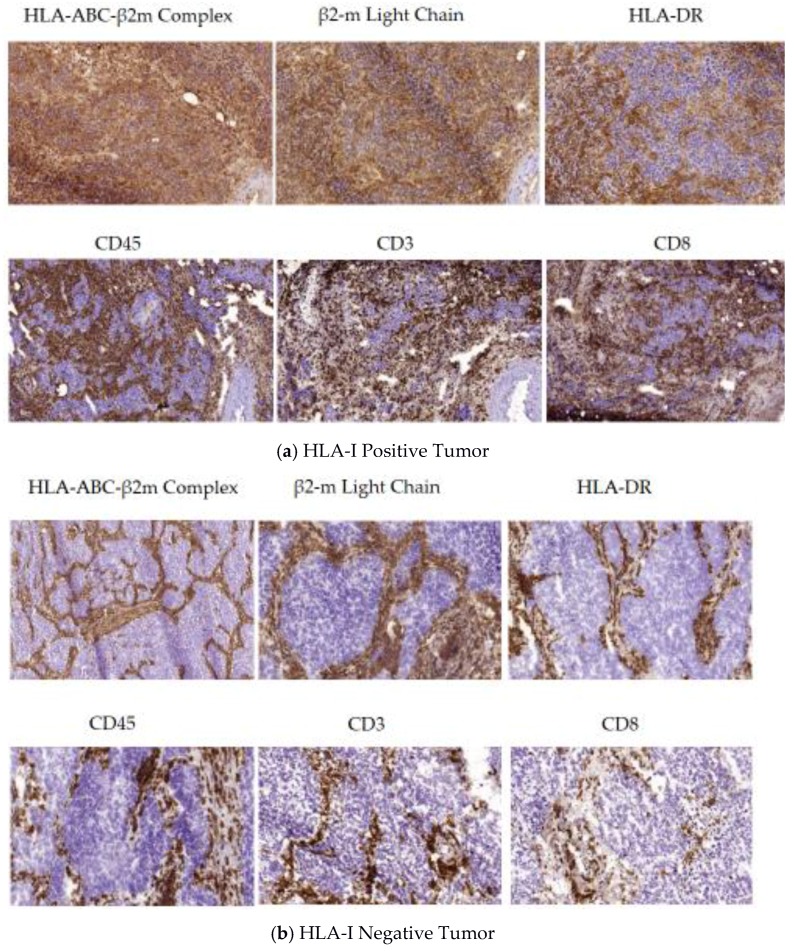
(**a**) Tissue architecture in HLA-I positive tumors (Phase I). Tumor tissue samples obtained from a patient diagnosed with non-small cell lung cancer (NSCLC) were immunostained with monoclonal antibodies against HLA-I, β2-microglobulin, HLA class II, CD8, CD3, and CD45 molecules. Most of the cancer cells are HLA-I positive and remain in close contact with HLA-I positive immune cells. The tissue structure is “permissive”, allowing TILs to enter the tumor mass, producing a significant CD8+ infiltration, and permitting a direct contact with cancer cells. Tumor parenchyma and stroma cannot be distinguished when immunostained for HLA-I expression. This tissue organization pattern in HLA-I positive tumors differs from that observed in HLA-I negative tumors depicted in Figure 2b; (**b**) Tissue architecture in HLA-I negative tumors (Phase II). In contrast to Figure 2a, this lung cancer tissue, obtained from another patient, is negative for both HLA-I and -II. T cells and other leukocytes are restricted exclusively to the peritumoral stroma that surrounds the tumor nest in a “non-permissive” tissue structure. There is a clear separation between tumor parenchyma and surrounding stroma. Tumor nodes are composed exclusively of HLA-I negative tumor cells without any infiltrating immune cells (phase of “immunological silence”).

When a tumor is HLA-I positive or heterogeneous, it is infiltrated with lymphocytes and macrophages owing to an immune-permissive tumor microenvironment (TME), in which tumor and TILs are in close contact with each other allowing recognition and destruction of the target tumor cells (Figure 2a). Tumors are infiltrated by CD4+ and CD8+ lymphocytes and by other leucocytes/macrophages at various degrees depending on the HLA-I positive/negative tumor cell ratio within the heterogeneous tumor mass. HLA-I expression was found to be correlated with intra- or peritumoral distribution of CD8+ T lymphocytes, which, in turn, is associated with different phases of tumor evolution, distinct tissue reorganization patterns and different M1/M2 ratios. HLA-I positive tumor cells are actively killed by CTLs in phase I, leading to a progressive T-cell immune selection of HLA-I deficient tumor cells (phase II).

### 5.2. Encapsulated Tumor Nodes Observed in HLA-I Negative Tumors (Phase II)

Phase II is characterized by the lack of tumor infiltrating cells and a clear separation between HLA-I negative tumor and HLA-I positive stroma cells. It is very likely that due to the low cytotoxic activity of CD8+ T lymphocytes and the absence of natural killer (NK) cells, in “phase II” the host is trying to isolate tumor cells from the rest of the body, while tumor cells are actively creating an immunosuppressive microenvironment by inducing stromal reorganization and generation of a structure similar to a TH2 granuloma [30]. As a result, tumor architecture changes, generating tumor nodes surrounded by different types of T-cells/leucocytes/macrophages and probably other elements of the tumor microenvironment, including Tregs and MDSCs. Furthermore, this phase is also characterized by a marked peritumoral localization of fibroblasts and alternatively activated macrophages providing a physical barrier and forming a “non-permissive” tissue structure (Figure 2b) [18]. Such tumor tissue organization can be observed in a variety of tumors of different histological type [8], however it has not been previously associated with the absence of HLA-I expression in tumors. Such an encapsulated HLA-I negative tumor without activated tumor infiltrating immune cells (phase II) represents a condition of “immunological silence”.

## 6. Progressive Exclusion and/or Inactivation of NK and CD8+ T Cells from the Distant Non-Tumor Tissue (DNTT) to the Tumor Tissue (TT)

**Figure 3:**
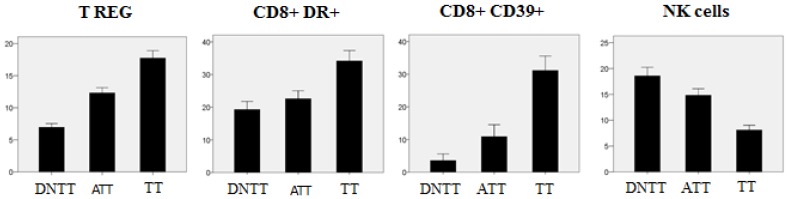
Variations in the percentage of lymphocyte subpopulations and natural killer (NK) cells in different areas of tumor and “normal” adjacent tissues measured by flow cytometry. DNTT—distant non tumor tissue, ATT—adjacent tumor tissue, TT—tumor tissue. The treg cell subset was defined as CD127low CD25bright CD4+. The number of Treg was calculated as a percentage of total number of CD4+ cells, while the CD8+ DR+ and CD8+CD39+ cell levels are shown as a percentage of CD8+ cells. NK cells were determined by the selection of CD45+ CD3− CD20− cells in the FSC^low^/SSC^low^ gate [31]. Tregs and CD8+CD39+ lymphocytes show increased presence closer to the tumor nest, while the percentage of NK cells is lower in TT as compared to other areas.

We have observed significant gradual changes in the composition of the inflammatory infiltrate in three different areas of cancerous lung tissue: tumor tissue (TT), tissue adjacent to the tumor (TAT), and distant non-tumor tissue (DNTT) [31]. Using a combination of flow cytometry and tumor tissue immunohistology, we found a considerable prevalence of activated CD8+DR+ T cells of effector-memory phenotype in tumor tissues (TT) as compared to TAT and DNTT areas. At the same time, we observed a remarkable gradual increase in the numbers of T-reg cells from the DNTT area towards TT (Figure 3) along with a reduction of the CD8/Treg and Th1/Treg ratio in tumor tissue as compared to a healthy counterpart. The anti-tumor CD8+ T cell-mediated cytotoxicity may be insufficient due to the increased presence of Treg cells and high frequency of CD8+ cells expressing CD39 (a marker associated with immunological exhaustion and paracrine inhibition of IFN-γ). In fact, CD39+ cells have been found to be highly represented in both CD4+ and CD8+ subpopulations in tumor tissues and were linked to immunosuppressive functions [32]. We favor the idea that the most important factor reducing the cytotoxic potential of CD8+ T-cells is associated with the lack of a “direct contact” with HLA-I negative target tumor cells due to an exclusive localization in the “stroma”, which creates a physical barrier between the HLA-I negative tumor cells and the infiltrating leucocytes/macrophages (see Figure 2b). In our study, using flow cytometry analysis of the cellular suspension obtained from the lung tumor samples, we observed that increase in the level of tumor infiltrating lymphocytes correlates with the increase in the proportion of Tregs (*p* = 0.012, R = 0.443) and CD8+CD39+ immunosuppressive cells (*p* = 0.015, R = 0.489) in this infiltrate. CD39 is an ectoenzyme, which serves as an integral component of the suppressive machinery of Tregs, inactivating and converting extracellular ATP into adenosine and allowing the immune escape of tumors. However, the high level of CD39 expression in both, CD4+ and CD8+ T cells, causes inhibition of the adhesion molecule expression necessary for transendothelial migration into the tumor and may explain the lack of infiltrating T-CD8 cells in the tumor nests [33].

**Figure 4:**
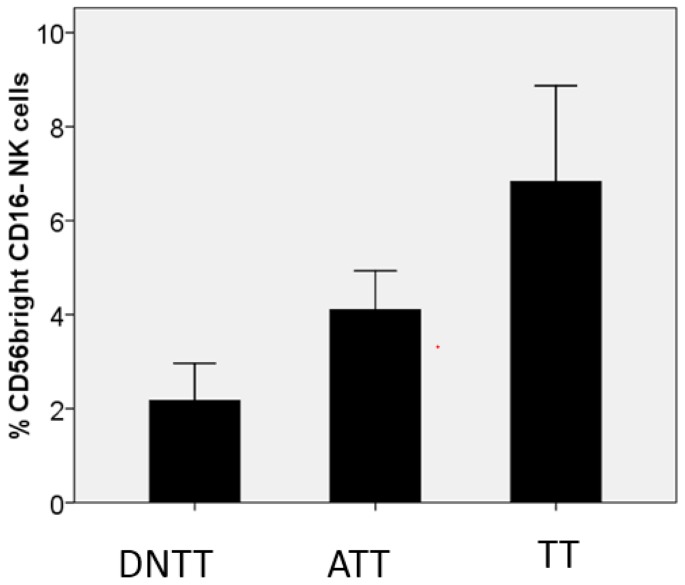
Changes in the percentage of CD56bright CD16− NK cells with impaired cytotoxic activity in different areas of the tumor and “normal” adjacent tissues analyzed by flow cytometry. TT is enriched by CD56bright CD16− NK cells, the percentage of which gradually increases from DNTT to TT. DNTT—distant non tumor tissue, ATT—adjacent tumor tissue, TT—tumor tissue. Tumor samples were obtained from primary lung tumors of non-treated patients by excision of a tumor mass fragment. ATT samples were taken from tumor-adjacent lung tissue located at approximately 1 cm from the periphery of the tumor without macroscopic signs of a tumor. DNTT and ATT samples were thoroughly analyzed to guarantee the complete absence of epithelial tumor cells. The NK cell subset was determined by the selection of CD45+ CD3− CD20− cells in the FSC^low^/SSC^low^ gate and calculated as a percentage of all NK cells [31].

With regards to NK cells, in the same study [31] we observed a significant decrease in the total number of these cells in tumor tissue (TT) as compared to the tissue adjacent to the tumor (TAT) or distant non-malignant tissue (Figure 3). On the other hand, the TT area is enriched with the NK cell subpopulation with the CD56bright CD16− phenotype and reduced cytotoxic activity (Figure 4). Altogether, the obtained results indicate that tumor nests are poorly infiltrated with NK cells, and even the small percentage of these cells detected within the tumor mass do not seem to have a cytotoxic potential to reject a tumor. Similar enrichment with CD56bright CD16− NK cells has been reported in NSCLC tumors [34]. Prevalence of the CD56+ CD16− NK subset with pro-angiogenic activity favoring tumor development and increased expression of inhibitory receptors in tumor infiltrating NK cells was reported in other types of cancer [35,36]. Lung adenocarcinoma cells frequently lose HLA-I expression [18] and theoretically should be heavily infiltrated by NK cells capable of eliminating HLA-I negative tumor cells. However, NK cells are rarely detected in the lung tumor infiltrate. Our findings point to the existence of alternative immune escape mechanisms influencing NK cells, such as (a) difficulty of homing, which translates to the practical exclusion of NK cells by cancer cells; (b) a progressive alteration in the phenotype of NK cells from healthy tissue to tumor tissue, with the emergence of a non-cytotoxic phenotype; in tumor tissue, we found a prevalence of CD56 bright CD16 negative non-cytotoxic cytokine-producing NK cells and detected non-cytotoxic CD56/CD16 negative cells with immature phenotype; and (c) the location of scarce NK cells in the tumor stroma without a direct contact with cancer cells (particularly in HLA-I negative tumors). Altogether, these results suggest that the observed changes are induced by the tumor microenvironment which locally impairs NK homing and differentiation, rendering these cells less cytotoxic and favoring the immune escape of HLA-I negative tumors.

## 7. Conclusions

There is accumulating evidence that tumor immune escape associated with MHC/HLA-I loss is a frequent phenomenon observed in human and experimental tumors. We have recently obtained data suggesting that the location of tumor infiltrating lymphocytes (TILs) in humans is associated with a particular tumor tissue organization. At the beginning of the T cell-mediated immune response, CD8+ T lymphocytes enter the tumor tissue and destroy HLA-I positive tumor cells (a permissive Phase I). Later, as a result of T cell immune selection, HLA-I negative tumor cell variants appear, ultimately generating a tumor composed only of HLA-I negative cells (Phase II). In this phase T lymphocytes and other leucocytes/macrophages reside outside the tumor nest. Such tissue structure provides a biological barrier between the tumor and stroma (a non-permissive Phase II). This well-established “tumor-parenchyma”-stroma architecture has been identified decades ago, but has never been linked to tumor HLA-I loss. We favor the idea that this reorganization of tumor tissue structure during cancer development is associated with the T-cell immune selection of MHC-I negative tumor cell variants and probably can be observed frequently since the percentage of human tumors with HLA-I loss reaches 65%–90% in different types of malignancy [8]. These figures are probably underestimated due to the difficulties in detailed analysis of HLA expression in tumor tissues. The expression of HLA-I molecules in the tumor cell surface could play a leading role in directing and conducting the TH1 response until the total elimination of transformed cells. In some patients, the appearance of HLA-I negative tumor variants as a result of immune escape from T cell responses may generate tumors composed only of HLA-I negative cells lacking tumor infiltration with immune cells and encapsulated by the stroma (“immunological silence”). The status of MHC/HLA-I expression in tumor samples could represent the “driving force” that determines tumor evolution: either a TH1-mediated tumor rejection or formation of TH2-type granuloma. The recovery of HLA-I expression in tumors is a major task for the future, since MHC/HLA molecules are playing a key role in antigen presentation and tumor rejection [12,37,38].

## Abbreviations

The following abbreviations are used in this manuscript:

HLA	human leukocyte antigen
β2m	beta-2-microglobulin
CTL	cytotoxic T lymphocyte
IFN	Interferon
LOH	loss of heterozygosity
TIL	tumor infiltrating lymphocyte
TME	tumor microenvironment
NSCLC	non-small-cell lung carcinoma
MHC	major histocompatibility complex
